# Understanding the association between unmet dental care needs and household food security status among older people in Ghana

**DOI:** 10.1186/s12903-023-03019-6

**Published:** 2023-05-25

**Authors:** Daniel Amoak, Joseph Asumah Braimah, Williams Agyemang-Duah, Yujiro Sano, Nancy Osei-Kye, Florence Wullo Anfaara, Roger Antabe, Ebenezer Dassah

**Affiliations:** 1 Department of Geography and Environment, Western University, London, Canada; 2 Department of Health & Society, University of Toronto Scarborough, Toronto, Canada; 3 Department of Geography and Planning, Queen’s University, Kingston, Canada; 4 Department of Sociology, Nipissing University, North Bay, ON Canada; 5 Department of Gender, Sexuality, and Women’s Studies, Western University, London, Canada; 6 School of Public Health, Kwame Nkrumah University of Science and Technology, Kumasi, Ghana

## Abstract

The literature recognizes food insecurity as a barrier to access to health care services. However, we know very little about the association between food insecurity and unmet dental care needs among older people in Ghana. To address this void in the literature, this study uses a representative survey of adults aged 60 or older from three regions in Ghana to examine whether older people who experienced household food insecurity differently report unmet dental care needs in comparison to their counterparts without any food insecurity. We find that 40% of older adults reported unmet dental care needs. Results from logistic regression analysis show that older people who experienced severe household food insecurity were more likely to report unmet dental care needs, compared to those who did not experience any type of food insecurity, even after accounting for theoretically relevant variables (OR = 1.94, p < 0.05). Based on these findings, we discuss several implications for policymakers and directions for future research.

## Introduction

Defined as the “limitation or uncertain availability of nutritionally adequate and safe foods or limited or uncertain ability to acquire food in socially acceptable ways” [1] (p.2330), food insecurity is a persistent public health issue facing older adults in sub-Saharan Africa (SSA) including Ghana [2]. About 66% of people in SSA experienced food insecurity in 2020, which was considerably higher than estimates in other world regions such as Central and Southern Asia (43%) as well as Latin America and Caribbean (41%) [3]. Among people within SSA, research suggests that older people are more likely to experience food insecurity in comparison to their younger counterparts [4]. This trend seems to be extended to older people in Ghana. For example, Gyasi et al. [5] find that 44% and 9% of people aged 50 or older experienced moderate and severe food insecurity, respectively. Blankson and Hall [6] also find among women aged 60 and older that 42% and 20% missed a meal the day before and went to bed hungry, respectively.

Research identifies food insecurity as a critical barrier to health care utilization. For instance,  it has been found in the United States that people who experience food insecurity, compared to their food-secure counterparts, were less likely to have a usual source of care and more likely to postpone needed medical care and medication [7]. Bhargava and Lee [8] also observe in the United States that food-insecure older adults are less likely to have a family physician or receive home health visits compared to those who do not experience food insecurity. Similar findings have been shown in low- and middle-income countries. In Indonesia, for example, food insecurity was also observed as a risk of restricted access to modern health care for outpatient care [9]. Research also shows in the Democratic Republic of Congo that the odds of adhering to antiretroviral therapy were lower among patients with food insecurity in comparison to those without any food insecurity [10].

Although these studies are useful, we know very little about the relationship between food insecurity and dental care utilization among older people in SSA including Ghana. This void in the literature may be problematic, considering that evidence shows food insecurity as a risk factor of poor oral health. For example, it has been shown that adults aged 60 or older with food insecurity are more likely to have untreated tooth decay, compared to their counterparts with food security [11]. Similarly, poor self-reported oral health is more prevalent among older adults with food insecurity [12]. In Ghana, Amoak et al. [13] find that older adults who report experiencing mild, moderate, and severe food insecurity are more likely to report poor oral health compared to those who were food secure. As food insecurity has been identified as a barrier to the utilization of health care services, it is concerning that older adults who experience food insecurity may be facing a unique barrier to dental care utilization in Ghana, despite their possible exposure to heightened oral health risks.

The oral health sector in Ghana is experiencing growth, but there is little investment in both private and public practice [13–15]. The sector’s underdevelopment is evident in the low ratio of dental practitioners to patients, estimated at 1 in 104, 000 [16, 17]. The dental sector is further characterized by spatial inequality whereby 75% of dentists operate in urban areas, with an estimated 70% situated in the two largest cities in the country, that is Accra and Kumasi [15, 17]. Reports further indicate that less than 30% of government hospitals and clinics have dental facilities, of which a third are ill-equipped or dilapidated [14]. While the National Health Insurance Scheme (NHIS) covers various oral health services such as pain relief, tooth extraction, dental restoration, and fillings [18], these services are also unevenly accessible [15, 19]. Specifically, inequities in healthcare access in Ghana, including low enrollment of the NHIS, shortage of personnel, unavailability and ill-equipped health facilities and an inability to pay for services, may compel vulnerable groups such as older adults to often delay visiting a dentist to address oral health problems, or leave them untreated [20]. This problem of unmet oral care is compounded by the unavailability of procedures for detecting, managing, and treating oral diseases in government-run primary healthcare facilities, such as the community health planning and services (CHPS) compounds that are prominent in rural areas of Ghana. These include inadequate screening for early detection of oral diseases and the lack of basic restorative dental procedures to treat existing dental decay [14].

While oral health is linked to social, psychological, and economic well-being, research also shows that oral health is systemically linked to chronic diseases such as diabetes and heart disease [21]. In this context, it is important to highlight that about 400 million people in SSA have been affected by oral health issues, thereby receiving a growing attention as a public health concern. This situation may be particularly relevant among older people in Ghana where the prevalence of caries and periodontitis is higher among those aged 55 years or older [16]. To this end, the current study uses a representative survey of adults aged 60 or older from three regions of Ghana to examine the association between unmet dental care needs and household food security status.

## Data

This study used data from a cross-sectional survey of older adults (people aged 60 years and over) in Ghana. Data were collected from June to August 2019 with the assistance of experienced research assistants from the University of Ghana, the University for Development Studies, and the Catholic University College, Ghana. Research assistants were trained on the survey and various ethical issues and were each made to sign a confidentiality agreement prior to the data collection. Ethical approval for the study was obtained from the Queen’s University General Research Ethics Board (GGEOPL-277-19). In addition, verbal and written informed consent was sought from the participants. Legally Authorized Representatives of illiterate participants (e.g., family members) also provided informed consent for the study. The data were collected using a multi-stage sampling framework. We randomly sampled one region each from three major agroecological zones in Ghana, enabling us to select three regions including Greater Accra Region from the Coastal Zone, Bono East Region from the Forest Zone, and Upper West Region from the Savannah Zone. Furthermore, we randomly drew two districts from each region making 6 districts in all. Ten enumeration areas were then randomly sampled from each district. Finally, we identified and interviewed one respondent each from randomly chosen households within these enumeration areas. Standardized data collection instruments on food security, health, health behaviours, and access to care were adapted from the Ghana Living Standards Survey [22] and the WHO Study on Global Ageing and Adult Health [23]. The total sample is 1,073. However, for the purpose of this study, we restricted the sample to 352 older adults who had any problems with mouth and teeth in the last 12 months.

### Dependent variable

Among respondents who had any oral and dental problem in the last 12 months, the survey asked whether they received any treatment from a dentist or other oral health specialist, with yes, no or can’t remember as possible responses. Based on this question, we created the binary dependent variable called ‘unmet dental care needs’ where they were coded as ‘unmet’ when they did not receive any treatment (0 = met; 1 = unmet).

### Independent variable

Food security status is our focal explanatory variable, which is constructed using the nine-item Household Food Insecurity Access Scale (HFIAS) developed by Coates et al. [24]. Respondents were asked to answer questions about the frequency of occurrence of each food insecurity situation on a Likert scale (0 = rarely; 1 = sometimes; 2 = often) when they experienced each situation Based on the scores from the HFIAS questionnaire, households were divided into four categories of food insecurity: food secure (i.e., scores 0–1), mildly food insecure (i.e., scores 2–7), moderately food insecure (i.e., scores 8–14), and severely food insecure (i.e., scores 15–27). Considering that the number of respondents who experienced mild insecurity was very small, we combined this category with moderate insecurity, resulting in that we have three categories for the dependent variable (0 = food security; 1 = mild/moderate insecurity; 2 = severe insecurity).

### Control variables

The relationship between household food security status and unmet dental care needs may be confounded by other factors. To account for potential founding factors, we introduced predisposing and enabling factors as control variables informed by the Andersen healthcare utilization model [25]. We added predisposing factors such as place of residence (0 = urban; 1 = rural), gender (0 = male; 1 = female), education (0 = secondary/higher education; 1 = primary education; 2 = no education), marital status (0 = currently married; 1 = formerly married; 2 = never married), age (0 = 80+; 1 = 70–79; 2 = 60–69), and religion (0 = Muslim; 1 = Christian; 2 = traditionalist; 4 = no religion). We included two enabling factors, namely health insurance enrolment (0 = yes; 1 = no) and household wealth (0 = richest; 1 = richer; 2 = poorer; 3 = poorest). Household wealth quintiles are measured by utilizing a composite index based on the presence of various household assets such as a vehicle, television, tractor, refrigerator, mobile phone, hoe, and radio.

### Statistical analysis

There were two separate analyses for this study. First, we employed univariate analysis to understand the characteristics of analytical sample. Second, due to the binary nature of the dependent variable, logistic regression analysis was applied to understand the relationship between household food security status and unmet dental care needs. Given the multi-stage sampling strategy, our analyses were weighted with sampling weights constructed for each stage of sampling based on the probabilities of each sampling unit being selected as part of the sample. Models were built sequentially. We explored the bivariate association between food security status and unmet dental care needs in Model 1 while Models 2 and 3 further accounted for predisposing and enabling factors, respectively. Results were shown with odds ratios (ORs). ORs larger than 1 indicate that people were more likely to experience unmet dental care needs while those lower than 1 have lower odds of doing so.

## Results

Table 1 shows sample characteristics. We find that 40% of older people with dental problems had unmet dental care needs. In addition, about half of the sample experienced severe food insecurity (49%) while slightly more than one-quarter did not experience food insecurity (28%). We also find that about half of the sample lived in rural areas (55%) and were female (48%), currently married (48%), and Christian (56%). About one in three older people had secondary or higher education (32%) although 44% did not have any formal education. It is also noteworthy that 76% of the sample did not have health insurance.

Table 2 shows findings from logistic regression analysis. In Model 1, we find at the bivariate level that older people who experienced severe food insecurity had higher odds of reporting unmet dental care need, compared to their counterparts who did not experience any food insecurity (OR = 2.08, p < 0.01). This significant relationship remained largely robust, even after accounting for predisposing factors in Model 2 (OR = 2.07, p < 0.05) and enabling factors in Model 3 (OR = 1.94, p < 0.05).

**Table 1:** Sample characteristics

	Percentage
Unmet dental care needs	
Met	60
Unmet	40
Food security status	
Secure	28
Mild/moderate	23
Severe	49
Place of residence	
Urban	45
Rural	55
Gender	
Male	52
Female	48
Education	
Secondary/higher	32
Primary	24
No education	44
Marital status	
Currently married	48
Formerly married	43
Never married	9
Age	
80+	30
70–79	29
60–69	41
Religion	
Muslim	33
Christian	56
Traditionalist	4
No religion	7
Household wealth	
Richest	25
Richer	24
Poorer	25
Poorest	26
Health insurance enrolment	
Yes	23
No	76
Total	352

**Table 2:** Logistic regression analysis of unmet dental care needs among elderly in Ghana

	Model 1		Model 2		Model 3	
	OR	SE	OR	SE	OR	SE
*Food security status*						
Secure	1.00		1.00		1.00	
Mild/moderate	1.72*	0.54	1.57	0.56	1.65	0.61
Severe	2.08***	0.56	2.07**	0.65	1.94**	0.64
*Place of residence*						
Urban			1.00		1.00	
Rural			1.56*	0.40	1.48	0.40
*Gender*						
Male			1.00		1.00	
Female			3.84***	1.02	4.13***	1.15
*Education*						
Secondary/higher			1.00		1.00	
Primary			1.25	0.44	0.80	0.31
No education			2.62***	0.85	1.44	0.54
*Marital status*						
Currently married			1.00		1.00	
Formerly married			1.06	0.29	0.98	0.27
Never married			2.45*	1.12	2.57**	1.21
*Age*						
80+			1.00		1.00	
70–79			0.91	0.31	0.87	0.31
60–69			1.96*	0.67	1.77	0.62
*Religion*						
Muslim			1.00		1.00	
Christian			1.54	0.45	1.72*	0.53
Traditionalist			4.08***	2.04	4.42***	2.34
No religion			1.91	1.21	2.30	1.51
*Household wealth*						
Richest					1.00	
Richer					1.64	0.67
Poorer					1.46	0.61
Poorest					2.49**	1.02
*Health insurance enrolment*						
Yes					1.00	
No					2.60**	0.99
F	3.74**		4.05***		3.51***	

*p < 0.1, **p < 0.05, ***p < 0.01; OR = odds ratio; SE = standard error

In addition to household food security status, a range of predisposing and enabling factors were significantly associated with unmet dental care needs (see Model 3 of Table 2). For example, female older people were more likely to report unmet dental care needs in comparison to their male counterparts (OR = 4.13, p < 0.01). Similarly, older people who have never been married were more likely to report unmet dental care needs in comparison to those who were married at the time of the survey (OR = 2.57, p < 0.05). In addition, older people who were affiliated with traditional religions were more likely to report unmet dental care needs, compared to their Muslim counterparts (OR = 4.42, p < 0.01). Compared to their richest counterparts, older people whose household wealth belonged to the ‘poorest’ category were also more likely to report unmet dental care needs (OR = 2.49, p < 0.05). Finally, older people without any health insurance had higher odds of reporting unmet dental care need in comparison to those who did (OR = 2.60, p < 0.05).

## Discussion and conclusions

Older adults from food-insecure households are often exposed to heightened risks of oral health issues, which are systemically linked to chronic diseases such as diabetes and heart diseases. Access to dental care services for timely diagnosis and treatment is particularly important among this population. As a barrier to health care utilization in general, however, dental care utilization may be restricted due to food insecurity. Using a representative survey of people aged 60 or older from three regions of Ghana, the current study aims to advance the literature by examining whether food security status is associated with unmet dental care needs among older adults.

We find that household food insecurity is a significant predictor of unmet dental care needs. Specifically, having an “unmet dental need” in the context of our study refers to respondents who self-reported a dental health problem but did not seek treatment for it. Thus, older people who experienced severe food insecurity were more likely to report unmet dental care needs compared to their counterparts who did not experience any food insecurity, even after accounting for a range of predisposing and enabling factors. This finding is largely consistent with Giannoni and Grignon [26], observing that food insecurity is associated with higher odds of reporting lack of access to dental care in Canada. Similarly, it is found in the United States that adults with low food security were more likely to have unmet dental care needs as compared to their counterparts with full food security [27]. In the Ghanaian context, Amoak et al. [13] highlighted that older people who experienced severe food insecurity were more likely to rate their oral-health as poor, pointing to possible unmet dental needs. Although the association between dental care utilization and food security status is largely explored in high-income countries such as the United States and Canada, it is imperative that food insecurity as a potential barrier to meeting dental care needs may be extended to older people in the LMICs including Ghana.

There are potential reasons to explain higher odds of reporting unmet dental care needs among older people with severe household food insecurity in Ghana. As explained by Weiner et al. [27], food insecurity is situated within the wider social context of the food system, which encompasses factors such as the availability of fresh and nutritious food. Older individuals, for example, may encounter difficulties in accessing such foods, which can compromise their dental health and diminish the benefits that are typically associated with a healthy diet. Other studies have also highlighted that food insecure adults are more likely to consume poor quality diets [28] and employ coping strategies that may include lower intake of vegetables, fruits, dairy products, zinc, calcium and magnesium [29–31]. While this association is established, earlier studies such as Barret [32] posits that the concept of food security is complex and multi-dimensional as it goes beyond measures of financial poverty to capture other household shocks including periodic unemployment and loss of productive assets that reflect the social and structural vulnerabilities of individuals and households. Indeed, food security is not only argued to represent the social vulnerability of households but may unveil their heightened exposure to economic and political disenfranchisement [32–34]. Furthermore, it has been argued that households may not prioritize health care utilization including dental care utilization when basic resources required for survival such as food and shelter are not accessible [35]. In addition to treatment cost, the cost of travelling to dental care facilities may be perceived as an extra burden on meeting household basic needs, considering that dental care resources are largely scarce in many SSA countries, often requiring people to travel far to have access to treatment from dental care facilities [16]. Previous research also points to the possibility that food-insecure households actively seek for cheaper treatment options [36]. In other words, older people with dental problems may rely on other alternative, cost-effective, and home-based remedies, instead of receiving formal treatment from dental care facilities. These arguments largely underline possible pathways where older people with food insecurity may redistribute limited household resources through avoiding dental care utilization, which may be perceived as less essential for survival.

Although not the focus of this study, we also find that some predisposing and enabling factors were significantly associated with unmet dental care needs. For example, we find that marital status was associated with unmet dental care need, showing that never married people were more likely to report unmet dental care needs, compared to their currently married counterparts. This finding is consistent with previous research, which argues that marriage can serve as social capital that promotes desirable health-related behaviours including access to dental care services [37]. We also find that traditionalists were more likely to report unmet dental care needs in comparison to Muslims. This finding may be explained by Peprah et al. [38] who reveal that some traditionalists can use self-treatment prescribed by faith healers. Finally, we find that low household wealth and lack of insurance enrolment were positively associated with unmet dental care needs. These findings are consistent with previous studies, suggesting that there are unequal financial barriers to health care utilization including dental care utilization in Ghana [15].

Based on these findings, there are several policy implications. The literature highlights that food insecurity possibly makes it difficult for older people to spend scarce household resources on items that are often perceived to be less essential for survival including access to dental care services [13, 27, 28]. This possible mechanism may be useful for informing food-related policies to pay attention beyond nutritional values of food and introduce policy strategies that enable food-insecure households to flexibly allocate material resources, which may increase their chance of receiving required health care attention including dental care attention. From the perspective of health-related policies, however, it is equally critical to review and potentially revise the NHIS in Ghana. Specifically, although the NHIS’s focus is to protect socially and financially vulnerable groups from being excluded from access to health care including dental care, some important measures of vulnerability such as food insecurity are not integrated as part of the policy [33]. These policy suggestions may be helpful for reducing the burden of unmet dental care needs among older people.

There are some limitations to this study. For example, we use a cross-sectional survey. Therefore, our results are limited to statistical association and cannot claim any causal relationship. In addition, we rely on the self-reported indicator of unmet dental care needs. Considering that we do not have any clinical and biomedical indicators of oral health, the accuracy of dental care needs is unknown and unmeasurable. For this reason, unmet dental care needs may be underreported or overreported. Thirdly, limiting our sample to individuals who self-reported oral health issues may have underestimated the actual magnitude of oral health problems in Ghana, as some people may not be aware of their oral health issues. Prospective studies should move beyond self-reporting and incorporate clinical measures to obtain a more accurate assessment of oral health issues among older adults in Ghana. Furthermore, in community-level studies in Ghana where access to regular dental check-ups remain low, we recommend future studies to include dental examinations as part of community-level health surveys. Additionally, the data also do not include other oral health-related behavioural factors such as tooth brushing, which may have been a predictor of unmet dental care needs. To overcome these limitations, incorporating clinical measures of oral health, such as the index of decayed, missing, and filled teeth, untreated cavities, and salivary flow, should be considered in future research [39]. We also recommend future research to employ longitudinal analysis as well as in-depth qualitative analysis, which may be useful for deconstructing the underlying processes that inform unmet dental care needs among older people in Ghana.

## Data Availability

The data used and/or analysed during the current study are available from the corresponding author on reasonable request.
